# Long-Term Outcomes of Ultrasound-Guided Thread Carpal Tunnel Release and Its Clinical Effectiveness in Severe Carpal Tunnel Syndrome: A Retrospective Cohort Study

**DOI:** 10.3390/jcm13010262

**Published:** 2024-01-02

**Authors:** In Jong Kim, Jae Min Kim

**Affiliations:** 1 Department of Rehabilitation Medicine, Howareyou Rehabilitation Clinic, Seoul 06134, Republic of Korea; sportsrehab76@gmail.com; 2 Department of Rehabilitation Medicine, Incheon St. Mary’s Hospital, College of Medicine, The Catholic University of Korea, Seoul 06591, Republic of Korea

**Keywords:** carpal tunnel syndrome, ultrasonography, thread carpal tunnel release

## Abstract

Ultrasound-guided thread carpal tunnel release (TCTR) was proposed as an effective and safe surgical technique with faster recovery and fewer complications. This study was conducted to confirm the long-term outcomes after TCTR and verify its clinical effectiveness in severe carpal tunnel syndrome (CTS) for more insights into TCTR procedure. A total of 168 TCTR procedures were performed in 152 individual patients by two physiatrists during 36-month period. In an assessment of 82 hands, surgical outcomes of 2 years after TCTR could be obtained, and the grade 6 CTS group of 21 hands, classified as extremely severe grade by Bland’s classification, was compared with other severity groups (grade 1–5). The Boston Carpal Tunnel Syndrome Questionnaire (BCTQ) was used to assess surgical outcomes. No adverse events occurred in all cases including the case of severe CTS and anatomical variants. TCTR showed significant improvement in BCTQ scale within 1–2 weeks, which continued up to 2 years with no recurrence (*p* < 0.01). Although slower and more progressive than the other severity group, there was also significant improvement relative to the BCTQ scale around 4 weeks after procedure in the grade 6 CTS group (*p* < 0.05). With the familiarity of ultrasound, ultrasound-guided TCTR is an effective and reliable surgical treatment for CTS in long-term outcomes and in severe CTS.

## 1. Introduction

Carpal tunnel syndrome (CTS) is the most frequent of the compressive neuropathies in the upper extremity and defined by compression and/or traction of the median nerve at wrist level. Its prevalence is between 4 and 5% among the general population, particularly affecting individuals between 40 and 60 years of age [1].

The treatments for non-deficit forms of CTS are medication, a night-time immobilization brace, and corticoid infiltration. However, in the case of resistance to conservative treatment or deficit forms, surgical treatment is indicated to achieve a reduction in intra-tunnel pressure by sectioning the transverse carpal ligament (TCL) [2].

Open carpal tunnel release (OCTR) is most frequently performed surgical procedure with relatively favorable outcomes [3]. However, because an incision of 3–4 cm is made, cutaneous nerve injury, hypertrophic scar with tenderness, pillar pain, and a delay in return to work are known to occasionally occur [3,4]. Endoscopic carpal tunnel release (ECTR) was introduced to reduce these complications in the late 1980s and resulted in a lower incidence of scar-related complications and earlier return to work than OCTR, but the risk of median nerve injury by insertion of an endoscope into carpal tunnel, a steep learning curve and its significant setup time are drawbacks [4,5].

With the development of interventional procedures using high-frequency ultrasound, ultrasound-guided percutaneous carpal tunnel release (USCTR) using various dividing instruments was proposed to overcome these limitations of OCTR and ECTR [6]. Among the USCTR, the thread carpal tunnel release (TCTR) was first proposed by Danqing Guo and his colleagues [7], which can transect the TCL without skin incision, leaving only two needle punctures [8]. Compared to other methods of USCTR, TCTR has the advantages of not using a sharp dividing instrument, requiring no repetitive cutting motions, and less iatrogenic injury by using the hydrodissection technique [9].

The clinical effectiveness and safety of TCTR, as well as its faster recovery and fewer complications compared to OCTR and ECTR, were reported by our previous study and other groups [8,9,10,11,12,13], and TCTR was expected to become the more popular surgical procedure for the treatment of CTS gradually. Therefore, it is necessary to investigate what extent TCTR can be applied for the surgical treatment of CTS in various clinical setting. However, there is a relative paucity of data available with reference to the long-term results of TCTR and its clinical effectiveness in severe CTS.

The purpose of this study was to determine the scope of clinical application of TCTR procedure by confirming the long-term outcomes after TCTR over a period of 2 years and verifying its clinical effectiveness in severe CTS.

## 2. Materials and Methods

### 2.1. Participants

A total of 184 hands in 168 patients (>18 years old) with symptoms of CTS were enrolled in this study from 1 March 2020 to 28 February 2023. Clinical symptoms that allowed the suspicion of CST Symptoms were hand numbness, tingling sensation, pain or weakness within median nerve territory and positive signs with provocation tests, such as Tinel at wrist and Phalen. Ultrasonographic and electrophysiologic evaluation were conducted in all patients for confirming the diagnosis and classifying the severity of CTS. For more detailed analysis, Bland’s more subdivided classification was used [14]. All enrolled patients failed to conservative treatment for more than 3 months.

**Figure 1:**
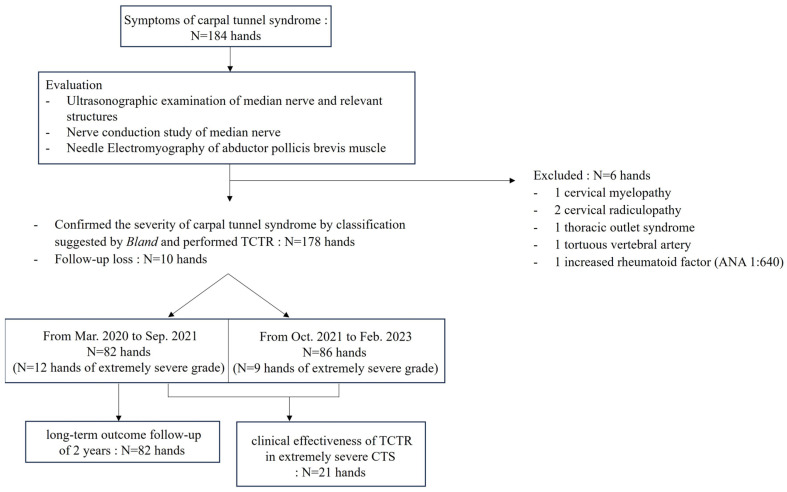
Flowchart detailing patient enrollment. TCTR, thread carpal tunnel release.

Six hands were excluded due to their comorbidities that could affect the result, such as cervical myelopathy, cervical radiculopathy, thoracic outlet syndrome, tortuous vertebral artery and increased rheumatoid factor. Because of a follow-up loss of ten hands after TCTR, the study included 168 hands in 152 individual patients (Mean age = 55.8 years, standard deviation [SD] ± 12.6 years, male: female = 53:115, 93 right-handed) (Figure 1).

The classification of CTS by the result of electrophysiologic findings was as follows: very mild 7 hands (grade 1, 4.2%), mild 10 hands (grade 2, 6.0%), moderate 57 hands (grade 3, 33.9%), severe 30 hands (grade 4, 17.9%), very severe 43 hands (grade 5, 25.6%), and extremely severe 21 hands (grade 6, 12.5%) according to Bland’s classification [14]. Anatomical variants were also evaluated in all patients who underwent TCTR before surgical procedure. Identified anatomical variants on ultrasonographic evaluation were as follows: 11 hands (6.5%) of bifid median nerve with or without persistent median artery and 2 hands (1.2%) of ulnar side branching of recurrent motor branch (RMB).

### 2.2. Procedure

#### 2.2.1. Equipment

All surgical procedures were performed under local anesthesia without tourniquet by two experienced physiatrists in the clinical procedure room using an LOGIQ S7 ultrasound machine (GE Healthcare, Seoul, Republic of Korea) fitted with a ML6-15 (8 MHz) linear array transducer. Smartwire CTS (0.27 mm in diameter, Smartwire Inc., Seoul, Republic of Korea) was used as cutting thread and it was developed for the percutaneous dissecting thread technique with higher cutting force, better visibility on ultrasound, and easier handling by additional coating of thin titanium nitride [13,15].

#### 2.2.2. Preoperative Evaluation

Preoperative ultrasound evaluation was performed before surgical procedure in all participants. The presence of intra-tunnel space occupying lesion, such as ganglion cyst or intra-tunnel aberrant muscle, such as proximal origin of lumbricals was evaluated first. Then, the relevant anatomical structures were evaluated including the course of median nerve, third common digital nerve (TCDN), RMB, superficial palmar arch (SupPA), and distal end of TCL, which called “duck’s beak” due to its shape [16]. Finally, it was confirmed whether there were some anatomical variations that could affect the surgical procedure, such as hypertrophic abductor pollicis brevis (APB), ulnar side branching of RMB, bifid median nerve with or without persistent median artery, Berrettini communicating branch (BCB), variant of superficial palmar arch (SupPA), and aberrant hypothenar muscle, if present.

#### 2.2.3. Surgical Procedure

First, the entry and exit points were determined and marked on the skin. The entry point was placed at the middle of palm, where the 3rd ray and a horizontal line from the apex of the interdigital fold between the thumb and index finger crosses perpendicularly. The exit point was placed just medial to the palmaris longus tendon, 1–2 cm proximal to the distal wrist crease. After sterile preparation and draping, 1–2 mL of lidocaine without epinephrine was injected subcutaneously at the entry and exit points with 27G needle. Under the ultrasound guidance, another 27G 2-inch needle with 5 mL of normal saline was inserted at the entry point and advanced over the SupPA and under the distal edge of TCL through the superficial and deep palmar aponeurosis (PA) to open up the distal carpal tunnel space with hydrodissection then removed.

Following the pathway made by previous step, a pre-bent 18G Tuohy needle (20-degree angle 1.5 cm distal to the tip with bevel up and 20–25-degree angle 2.0 cm proximal to the tip) with 5 mL of normal saline was inserted at the entry point and advanced through the dorsal side of the TCL with additional hydrodissection. After the needle tip exited at the determined exit point, a cutting thread was passed through the needle. Leaving the thread at the dorsal side of the TCL, the needle was removed.

With continuous ultrasound guidance, another pre-bent 18G Tuohy needle (10-degree angle 1.0 cm distal to the tip with bevel up) with 5 mL of normal saline was inserted at the same entry point and advanced through palmar side of TCL. After the needle tip exited at the same determined exit point, the cutting thread left at the dorsal side of the TCL was passed through the needle distally then looped around the TCL by removing the needle.

Using the axial and sagittal sonographic view, the absence of RMB, TCDN, BCB, and SupPA within the loop of cutting thread was confirmed. Then, the TCL was dissected by moving the cutting thread reciprocally. After the removal of the thread, the patient’s dissected TCL was confirmed through ultrasound examination and RMB was checked immediately by asking thumb opposition.

After hemostasis by gentle compression, compressive dressing is applied with commercial wrist brace.

#### 2.2.4. Postoperative Instruction

All patients were allowed to commence the simple daily activities as tolerated immediately after procedure and instructed to remove the dressing by themselves and wash their hands in 3 days. Lifting heavy objects or clenching hands was prohibited until 2 weeks after surgery.

Participant’s occupation was classified into 3 categories: white-collar worker, blue-collar worker with light, repetitive hand motions, and blue-collar worker with heavy, repetitive hand motions, and return to work was permitted in 3 days, 2 weeks, and 3–4 weeks, respectively, as tolerated. However, there were no restrictions on any activities or return to work, if no pain.

### 2.3. Materials

The clinical effectiveness of TCTR was assessed by Boston Carpal Tunnel Syndrome Questionnaire (BCTQ), which is a validated patient-centered measure for quantifying the symptom severity and disability [13]. BCTQ scores range between 1 and 5, with 1 representing the no pain or no difficulty and 5 representing the most severe symptom or functional limitation. The BCTQ scores of pre-TCTR and 1 week after TCTR were measured in the clinic, and the BCTQ scores of 2 weeks, 4 weeks, 2 months, 3 months, 6 months, 1 year, and 2 years after TCTR were measured via google survey link or phone interview in most cases.

The follow-up period to determine the long-term prognosis after TCTR was set 2 years, because the mean time to revision surgery was 1.23 years after OCTR or ECTR [17].

Because of considerable differences among the post operative outcomes according to the severity, especially in grade 6 CTS [14], Bland’s classification was used for severity classification instead of Steven’s relatively simple one [18]. The effectiveness of TCTR in grade 6 CTS was evaluated separately from other grades, and the proportion of “unchanged” plus “worse” after TCTR was measured additionally in that grade.

### 2.4. Statistical Analysis

Medical records collected by two physiatrists were anonymized by separated data collector and then sent to the data analyzer. Statistical analysis was carried out using SPSS statistics version 22 (IBM, Armonk, NY, USA). The normality of the distribution was assessed by the Shapiro–Wilk test. The paired *t*-test and Wilcoxon signed-rank test were performed to analyze pain reduction and functional gain over time in each group. The independent *t*-test was performed to compare the grade 6 CTS group with other severity group (grade 1 to 5) at each outcome-measured period. Statistically significant level was set to *p*-value of <0.05.

## 3. Results

### 3.1. Long-Term Outcomes

A total 168 hands in 152 individual patients were included in this study and long-term outcomes over a period of 2 years could be evaluated in 82 hands. Their pre-TCTR BCTQ severity score was 3.21 (SD ± 0.82) and showed a significant improvement of 1.65 (SD ± 0.55) in 1 week (*p* < 0.01). This improvement continued progressively until 4 weeks (BCTQ severity score = 1.41, SD ± 0.55) and plateaued after that. At 1 year and 2 years after TCTR, BCTQ severity score showed 1.05, SD ± 0.07 and 1.03, SD ± 0.05, respectively, and was well maintained without worsening up to 2 years (Table 1).

Regarding hand function, the pre-TCTR BCTQ function score was 2.42 (SD ± 0.84) and showed a significant improvement of 1.76 (SD ± 0.56) in 2 weeks (*p* < 0.01). Additional improvements were shown until 3 months (BCTQ function score = 1.32, SD ± 0.34) and plateaued thereafter as the severity remained stable. At 1 year and 2 years after TCTR, BCTQ function score showed 1.08, SD ± 0.11 and 1.04, SD ± 0.08, respectively, and was well maintained without worsening up to 2 years (Table 1).

### 3.2. Clinical Effectiveness in Severe Carpal Tunnel Syndrome

Twenty-one hands of grade 6 CTS were analyzed separately to confirm the clinical effectiveness of TCTR in severe CTS.

Pre-TCTR BCTQ severity scores were 3.01 (SD ± 0.82) in the grade 6 CTS group and 3.25 (SD ± 0.78) in the other severity group. There was no significant difference in pre-TCTR BCTQ severity score between the two groups (*p* = 0.193). Both groups showed significant improvements in BCTQ severity score at 1 week after the TCTR of 2.04 (SD ± 0.69) and 1.60 (SD ± 0.51), respectively (*p* < 0.01) (Table 2).

**Figure 2:**
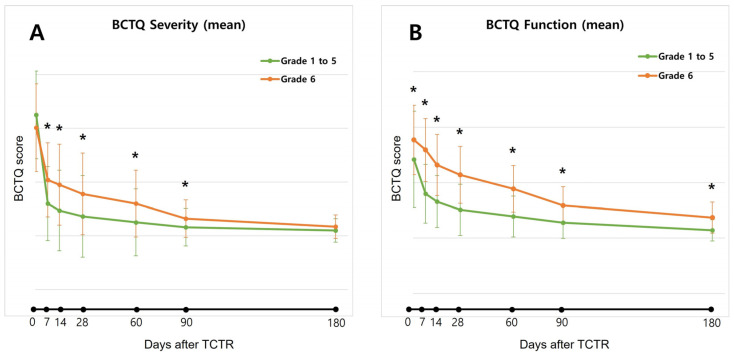
Post-TCTR time-dependent changes of patients in grade 6 CTS group and other severity group. (**A**) BCTQ severity scale and (**B**) BCTQ function scale. TCTR, thread carpal tunnel release; BCTQ, Boston Carpal Tunnel Syndrome Questionnaire. * Denotes significant difference between groups at each follow-up time by independent *t*-test, *p* < 0.05.

In contrast to the other severity group, where a notable improvement in the BCTQ severity score occurred within 1 week and plateaued by 4 weeks post-TCTR, the grade 6 CTS group exhibited comparatively less improvement during the first 1 to 4 weeks. The plateau for this group was reached around 3 months after TCTR, as illustrated in Figure 2A

Despite a slower and less distinct recovery compared to severity, hand function showed an overall similar pattern to severity and significant improvements in BCTQ function score were shown after TCTR.

Pre-TCTR BCTQ function scores were 2.77 (SD ± 0.63) in the grade 6 CTS group and 2.42 (SD ± 0.87) in other severity group. In contrast to BCTQ severity score, there was significant difference in pre-TCTR BCTQ function score between two groups (*p* = 0.029). Both groups showed significant improvements in BCTQ function score until 6 months as 1.37 (SD ± 0.28) and 1.14 (SD ± 0.19), respectively (*p* < 0.05 in the grade 6 CTS group, *p* < 0.01 in other severity group) (Table 2).

In the other severity group, a significant improvement in BCTQ function score was shown within 2 weeks and plateaued in 3 months after TCTR, but a much slower and progressively improving trend was observed throughout 6 months after TCTR in the grade 6 CTS group (Figure 2B).

The proportion of “unchanged” plus “worse” after TCTR in the grade 6 CTS group was 9.5% (2 in 21 hands).

### 3.3. Adverse Events and Others

No adverse events were reported, including nerve injury, vascular damage, or infection even in the case of anatomical variants, such as bifid median nerve or ulnar side branching of RMB. The surgical time from the first local anesthesia to the resection of TCL was within 20 min and more than 5 min of compression for hemostasis was not necessary in all cases. All patients could commence their activities of daily living and return to work according to the instructed schedules as follows: within 3 days in white-collar workers, 2 weeks in blue-collar workers with light, repetitive hand motions, and 3–4 weeks in blue-collar workers with heavy, repetitive hand motions after the procedure, except one case.

One patient suffered painful wrist swelling after the procedure and iatrogenic partial resection of accessory abductor digiti minimi (ADM) muscle was identified on a follow-up ultrasound examination. However, his symptoms subsided spontaneously without any invasive interventions, and he could resume the daily activities in 2 weeks. He was a video camera man with heavy, repetitive hand motions and returned to work in 5 weeks after TCTR without restrictions.

No patient was necessary to perform revision surgery except for one case. Nearly complete relief of symptoms was shown immediately after TCTR in that case, but relapse of symptoms occurred in 5 months after the procedure. Due to the patient’s preference, revision TCTR was performed again, and the patient had complete symptom relief without recurrence.

## 4. Discussion

Ultrasound-guided TCTR was proposed by Danquing Guo and colleagues first. They verified the clinical effectiveness and safety of TCTR and improved the technique of procedure through their cadaveric and clinical studies [7,8,9]. Other groups also reported faster clinical improvement in various parameters and a shorter return to work compared to OCTR and ECTR with fewer complications [10,11,12]. With these advantages, TCTR seems to play an important role for the surgical treatment of CTS in the future. However, there was relatively little evidence of long-term outcomes after TCTR and its clinical effectiveness in severe CTS. In this clinical perspective, the objective of this study was to confirm the long-term outcomes of a 2-year period after TCTR and verify its clinical effectiveness in severe CTS for more insights about the scope of its clinical application.

### 4.1. Current Surgical Technique

**Figure 3:**
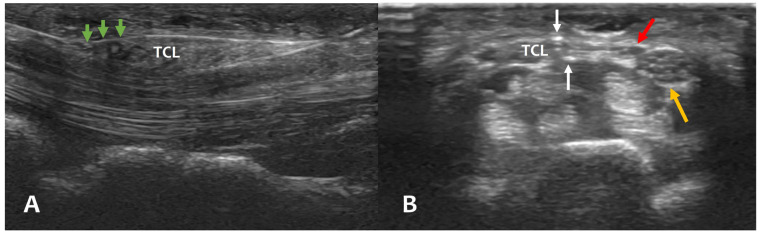
Realtime ultrasound images of thread placement during TCTR procedure. (**A**) Green arrows: thread placement along the fusiform proximal TCL (longitudinal view). (**B**) Yellow arrow: median nerve; red arrow: ulnar side branching of RMB; white arrows: thread placement medial to the ulnar side branching of RMB (axial view). TCTR, thread carpal tunnel release; TCL, transverse carpal ligament; RMB, recurrent motor branch.

The modified TCTR technique suggested by Danquing Guo and colleagues [8], which was also considered standardized technique in other groups [10,11,12,13], was used in this study. However, our technique differed from Guo’s modified TCTR technique with two minimal modifications. First, because the boundary between PA and TCL was unclear on ultrasound in some cases, especially at the proximal part of TCL, PA was included in the looped thread and not preserved in this study to avoid incomplete resection of TCL. However, there was no significant difference from other clinical studies of TCTR with PA preservation in pillar pain and recovery time to work [8,12,13]. Second, during the second Tuohy needle passage, which was palmar to TCL, needle-tip manipulation with bevel turning was used to locate the thread along the fusiform shape of proximal part of TCL (Figure 3A) and avoid unnecessary resection of aberrant hypothenar muscle, especially in the muscular type of accessory ADM [19]. After we experienced one case of self-limited painful wrist swelling caused by iatrogenic partial resection of accessory ADM, this modification was used to reduce post-operative pain and time to recovery.

### 4.2. Long-Term Outcomes

Long-term outcomes of the 2-year period could be evaluated in 82 hands. Because the mean time to revision surgery was 1.23 years after OCTR or ECTR [17], long-term follow up of 2 years was a reliable period for the evaluation of recurrence after TCTR.

As per previous clinical studies [8,9,10,11,12,13], the recovery process of TCTR was also much faster than that of OCTR or ECTR in this study. There were significant improvements in 1 or 2 weeks, and they continued progressively about 4 weeks and plateaued after that in both severity and function. Until 2 years after TCTR, these improvements were well maintained without recurrence of symptoms.

### 4.3. Clinical Effectiveness in Severe CTS

One of the purposes of this study is to determine whether TCTR can be performed effectively in severe CTS. Although the clinical effectiveness of TCTR in severe CTS was reported in some previous clinical studies [10,11,12,13], the classification proposed by Stevens, which divides the CTS simply into 3 grades as mild, moderate, and severe, was used in all of them. This classification may be useful to determine the proper treatment options according to the individual severity, but it cannot reflect degree of median nerve damage in CTS accurately.

Bland and his colleagues categorized the severity of CTS into 6 grades and evaluated the overall subjective outcomes after OCTR or ECTR using five-point rating scale: “cured”, “much better”, “better”, “unchanged” and “worse” [20]. According to the more detailed classification suggested by Bland, the severe grade proposed by Stevens was subdivided into severe (grade 4), very severe (grade 5), and extremely severe (grade 6) [14]. Interestingly, the surgical outcome in grade 6 CTS was much worse than that in grade 4 or grade 5. The proportion of “unchanged” plus “worse” after OCTR or ECTR in grade 6 was 31.7%, but only 12.2% in grade 4 and 13.7% in grade 5 [14]. Thus, Stevens’ classification was not suitable to evaluate the exact outcomes following carpal tunnel release in severe CTS. To verify whether TCTR can be performed effectively in severe CTS, it would be the better way to separately evaluate the outcome in grade 6 CTS alone, excluding grade 4 and 5, which have relatively favorable surgical outcomes. If the surgical outcomes of TCTR are favorable even in grade 6 CTS, TCTR can be proposed as an effective surgical treatment for CTS regardless of its severity.

Twenty-one hands of grade 6 CTS were included in the current study. Unlike the other severity group, which showed significant improvement within 1–2 weeks after TCTR, the BCTQ severity score took 2–4 weeks to improve and BCTQ function score improved gradually over 6 months in the grade 6 CTS group. Although there were transient exacerbations of symptoms in some cases, most patients of the grade 6 CTS group showed overall favorable outcomes in both severity and function within 6 months. The transient aggravation of symptoms was presented 1–2 weeks after TCTR, not immediately after procedure, then subsided in 1 to 2 months spontaneously. Before the procedure, practitioners should warn about the possibility of transient worsening of symptoms after TCTR and assurance of its benign course, especially to the patients of grade 6 CTS. This slow recovery and transient exacerbation of symptoms in that grade may be due to time-consuming nerve regeneration and resulting hypersensitivity. The increase in intratunnel pressure during the TCTR procedure also may be the potential cause of this transient exacerbation.

Although the number of grade 6 CTS included in this study was much smaller than that in Bland’s, as the two studies consisted of 21 hands vs. 483 hands, respectively [14], the proportion of “unchanged” plus “worse” after TCTR in the grade 6 CTS group was 9.5% (2 in 21 hands) without any “worse” cases, which was much smaller than 31.7%, as Bland reported. The two “unchanged” patients’ symptoms were not too severe to interfere their daily living, and they did not want an additional surgical procedure to be performed. Because their data were collected at 6 months and 1 year after TCTR, respectively, it seems to be relatively insufficient time period to confirm their overall recovery.

Although the recovery process was relatively slow and progressive, TCTR was effective and showed favorable outcomes even in grade 6 CTS. Thus, it was verified that TCTR can be performed effectively in most CTS regardless of its severity.

### 4.4. Considerations for Anatomical Variations

Major and digital nerve injuries were very rare in OCTR and ECTR [21] and also are not reported in the clinical studies of TCTR [8,9,10,11,12,13] including this study. However, due to the high variability of RMB with regard to its course in relation to TCL [22], RMB may be susceptible to iatrogenic injury during TCTR, especially in the type of ulnar side branching. However, because the incidence of ulnar side branching of RMB was very low, ranging from 0.9 to 3.6% [22], and its reliable visualization on high-resolution ultrasound [23], iatrogenic injury of RMB can be avoided through the thorough ultrasound evaluation of median nerve before and during TCTR. In this study, 2 cases (1.2%) of ulnar side branching of RMB were identified and performed TCTR without any complications (Figure 3B).

Another considerable variation of RMB is the transligamentous type with a pooled prevalence rate of 11.3% [22]. Because of the possibility of compression within the reticular fibers, isolated RMB neuropathy can occur in transligamentous type [24]. Because the pooled prevalence of transligamentous course was 23.4% in patients with hypertrophic APB, as compared to 1.7% without it, practitioners should consider the potential RMB variants in patient with hypertrophic APB. Although isolated RMB neuropathy was either rare or possibly underdiagnosed [23] and was not encountered in current study, OCTR seem to be indicated instead of TCTR in isolated motor deficiency because the exploration of RMB may be needed [2,25].

The digital nerves most likely to be damaged during TCTR are the TCDN of the median nerve and the BCB between the median and ulnar nerve, which is present in around 80% [26]. However, because of their clear visualization on high-frequency ultrasound and the hydrodissection technique, iatrogenic injury could be prevented during TCTR in this study. This holds true, irrespective of the presence or classification of the nerves, aligning with findings from other clinical studies [8,9,10,11,12].

The bifid median nerve, with a prevalence of 9–13% [27], can make the TCTR procedure difficult by narrowing the safety zone, which is the space between the median nerve and ulnar artery. We identified 11 cases (6.5%) with a bifid median nerve, which were not excluded but instead included in our study. These cases underwent TCTR without encountering any adverse events. Despite instances where a portion of the thread was positioned palmar to the ulnar-sided bifid median nerve, minimal movements along the long axis of the median nerve did not result in any damage. This was attributed to the smooth surface of the thread.

Although SupPA had 18.7% of incomplete variants [28], which was also encountered in our study, initial hydrodissection was performed successfully through just under the distal part of TCL in all cases.

### 4.5. Reoperation after TCTR

Another consideration is the possibility of reoperation after TCTR. The reported incidence of revision surgery was 1.4–4.4% in OCTR and 2.8–6.5% in ECTR [17,21]. Although the cumulated number of cases that underwent TCTR was much smaller than that of OCTR and ECTR, the pooled reoperation rate of TCTR was about 1.4% (7 in 497 hands) including one case of revision TCTR in current study [8,9,10,11,12,13]. Because the focal swelling of median nerve with an hourglass deformation was a prominent feature on follow-up ultrasound evaluation in that case, the incomplete release of TCL was thought to be a more reliable cause of recurrence rather than adhesion or scar tissue formation. Most cases of reoperation after TCTR were related to the incomplete division of distal TCL in early stage of learning curve. However, after the introduction of the currently standardized proximal to distal cut technique [11], or an additional step for the confirmation of complete TCL resection with a blunt, rigid cannula [12], there were no further cases requiring the revision surgery.

### 4.6. Another Considerations

Because the whole process of TCTR was performed under ultrasound guidance, the sufficient skill of handling and interpreting the ultrasound is essential. Contraindications of TCTR should include not only the indications of surgical exploration, but also the failure of identification of relevant anatomy and thread position on ultrasound. A significant learning curve is also required for successful outcomes [12]. Schrier et al. suggested that 25 and 50 cases of supervision are needed for the practitioner with and without previous ultrasound experience, respectively [29]. In the case of motor form only [25], intra-tunnel space occupying lesion, and acute CTS like median artery thrombosis, OCTR with exploration should be indicated rather than TCTR [1].

Depending on the medical system of each country, the use of ultrasound and disposable dissecting thread may increase the medical cost of TCTR procedure. However, because it does not require general or brachial anesthesia and hospitalization for postoperative pain control, TCTR can offset this increase in direct surgical cost. Furthermore, the patient’s early return to work with faster recovery after TCTR has the potential to reduce the overall socioeconomic burden of carpal tunnel release [9].

### 4.7. Limitations of the Study

The limitations of this study are that it was retrospective and used a relatively small amount of long-term follow up and grade 6 CTS. Well-designed, controlled randomized studies with direct comparison of TCTR to OCTR and ECTR for longer time periods using more patients are needed in the future.

## 5. Conclusions

Ultrasound-guided TCTR showed reliable and favorable outcomes in the long-term follow up. It was not only effective even in the CTS of extremely severe grades but also safe in CTS with anatomical variants. If the practitioner is familiar with ultrasound-guided procedures, it can be performed effectively and safely in most CTS regardless of its severity or presence of anatomical variants.

## Figures and Tables

**Table 1:** Long-term outcomes following TCTR.

Outcome	Pre-TCTR	1 Week Post	2 Weeks Post	4 Weeks Post	2 Months Post	3 Months Post	6 Months Post	1 Year Post	2 Years Post	*p*
Severity ^1^ (Mean, SD)	3.21 (0.82)	1.65 (0.55)	1.53 (0.56)	1.41 (0.55)	1.30 (0.41)	1.20 (0.29)	1.12 (0.18)	1.05 (0.07)	1.03 (0.05)	<0.01 ^3^
Function ^2^ (Mean, SD)	2.42 (0.84)	1.90 (0.60)	1.76 (0.56)	1.60 (0.52)	1.45 (0.43)	1.32 (0.34)	1.18 (0.23)	1.08 (0.11)	1.04 (0.08)	<0.01 ^3^

TCTR, thread carpal tunnel release. ^1^ Severity by BCTQ (1–5): 1 = no symptoms, 5 = maximum symptoms. ^2^ Function by BCTQ (1–5): 1 = normal, 5 = abnormal. ^3^ Paired *t*-test results in each follow-up interval.

**Table 2:** Outcomes following TCTR according to the severity.

Outcome	Hand	Pre-TCTR	1 Week Post	2 Weeks Post	4 Weeks Post	2 Months Post	3 Months Post	6 Months Post	*p*
Severity ^1^ (Mean, SD)	CTS of grade 6	3.01 (0.82)	2.04 (0.69)	1.95 (0.75)	1.78 (0.76)	1.60 (0.62)	1.32 (0.35)	1.17 (0.22)	<0.05 ^3^
	CTS of other grades (grade 1–5)	3.25 (0.78)	1.60 (0.51)	1.47 (0.51)	1.36 (0.50)	1.25 (0.36)	1.16 (0.24)	1.10 (0.17)	<0.01 ^4^
Function ^2^ (Mean, SD)	CTS of grade 6	2.77 (0.63)	2.59 (0.57)	2.32 (0.55)	2.14 (0.51)	1.89 (0.42)	1.59 (0.34)	1.37 (0.28)	<0.05 ^3^
	CTS of other grades (grade 1–5)	2.42 (0.87)	1.80 (0.53)	1.66 (0.47)	1.51 (0.46)	1.37 (0.37)	1.25 (0.28)	1.14 (0.19)	<0.01 ^4^

TCTR, thread carpal tunnel release; CTS, carpal tunnel syndrome. ^1^ Severity by BCTQ (1–5): 1 = no symptoms, 5 = maximum symptoms. ^2^ Function by BCTQ (1–5): 1 = normal, 5 = abnormal. ^3^ Wilcoxon signed-rank test results in each follow-up interval. ^4^ Paired *t*-test results in each follow-up interval.

## Data Availability

The data presented in this study are available on request from the corresponding author.
